# Morpho-physiolological and qualitative traits of a bread wheat collection spanning a century of breeding in Italy

**DOI:** 10.3897/BDJ.3.e4760

**Published:** 2015-08-14

**Authors:** Paolo Laino, Margherita Limonta, Davide Gerna, Patrizia Vaccino

**Affiliations:** ‡ Consiglio per la ricerca in agricoltura e l'analisi dell'economia agraria, Unità di ricerca per la Selezione dei Cereali e la Valorizzazione delle varietà vegetali (CREA-SCV), Sant'Angelo Lodigiano, Italy

**Keywords:** *Triticum aestivum*, phenotyping, genetic resources, Italy, wheat breeding

## Abstract

Evaluation and characterization are crucial steps in the exploitation of germplasm collections. The Sant’Angelo Lodigiano unit of the Consiglio per la ricerca in agricoltura e l’analisi dell’economia agraria (CREA) maintains a broad collection of *Triticum* spp, including more than 4000 genotypes of *T. aestivum*. Such collection represents a wide source of genetic variability for many agronomic and qualitative traits, extremely useful in modern breeding programs. The collection size, however, makes very difficult its management as a whole. A reduced subset, representing the process of wheat breeding in Italy during the last hundred years, was hence identified for an in-depth characterization. The lines were cropped in two locations over two growing seasons, and analyzed using 16 morpho-agronomic and qualitative descriptors. Most of the analysed characters showed a broad variation throughout the collection, allowing to follow the plant ideotype changes across the breeding progress in Italy during the 20th century.

## Introduction

Wheat is one of the most important crop worldwide, occupying 17 percent of the total cultivated land in the world and providing the staple food for 35 percent of the world’s population (http://www.idrc.ca/EN/Resources/Publications/Pages/ArticleDetails.aspx?PublicationID=565). In Italy, during 2013, wheat was cultivated on approximately 1.9 million hectares, 632,000 of which devoted to bread wheat, the remaining to durum (ISTAT 2013).

Although wheat breeding started some years later in Italy than in other European countries such as Germany, France and Sweden, a great effort was soon dedicated to it. As far as bread wheat is concerned, at the beginning of the 20th century its cultivation in Italy relied mainly on landraces (Gentil Rosso, Cologna, Rieti, Solina) and some French genotypes (i.e. Inallettabile); breeding consisted only in the selection of their best variants. Nazareno Strampelli (1866-1942), rightly considered the pioneer of wheat breeding in Italy, started his remarkable career by selecting the best genotypes from the rust-resistant wheat landrace “Rieti”. Nevertheless, despite his early success, he quickly realized that it was difficult to obtain significant improvement by selecting only within population. Unaware of Mendel’s laws of inheritance (rediscovered only in 1900 by De Vries, Correns and Von Tschermak), he started a deliberate crossing program involving Italian and foreign varieties (Strampelli 1907). His first bread wheat release, Carlotta Strampelli, was soon followed by many others, including three cultivars (Ardito, Mentana and S. Pastore) which played a key role in Italian and international wheat improvement. Strampelli’s wheats allowed Italy to increase crop production during the so called “Battaglia del grano" (Battle for Grain; 1925-1940), launched by the Italian government in order to achieve self-sufficiency in wheat production; furthermore, several of them became part of the pedigree of many outstanding varieties from all over the world (i.e. Mexico, Brasil, Canada, USA, Argentina, Jugoslavia, Russia, Greece, Albany and China) (Borojevic and Borojevic 2005).

Once the superiority of hybridization over within-population selection was established, wheat breeding was carried on using hybridization. This led to the constitution of several important cultivars. In the 50s and 60s cvs S. Pastore, Autonomia, Mara and Funo dominated the seed market, S. Pastore being the most widespread cultivar until mid-70s. The 70s saw Marzotto and Irnerio taking the leading positions, while in the next decade Centauro, Pandas and Mec got the leadership of the market. Centauro, in particular, was the most diffused cultivar until 2000, when it was displaced by Serio. Serio, together with Bolero and Mieti, had an outstanding spread until the half of the first decade of the 21st century, when a quicker turnover, mainly based on foreign varieties, started to be observed. Nowadays the six most cultivated varieties (Bologna, Aubusson, Blasco, PR22R58, Altamira and Solehio) cover more than 30% of the total area, according to estimates based on the distribution of certified seed, which represent more than 90% of the seed annually sold in Italy.

CREA-SCV maintains a collection of *Triticum* sspp. including more than 4000 genotypes of *T. aestivum*. Such collection represents a wide source of genetic variability for many agronomic and qualitative traits, extremely useful in modern breeding programs. Thanks to the funding of the project RGV-FAO (started in 2004 in order to implement the FAO International Treaty on Plant Genetic Resources for Food and Agriculture) financed by the Italian Ministry of Agriculture (MIPAAF), the collection is maintained, catalogued and characterized by passport and specific descriptors, such as pedigree, country of origin, plant height, glume and seed colour, etc. The huge size of the collection, however, makes very difficult its management as a whole; as a consequence, we decided to focus on a reduced subset made of 226 accessions representing the bread wheat breeding process in Italy during the last hundred years. The evaluation of the genetic variation of such collection for several morpho-physiological and qualitative traits is the objective of the present study.

## Material and methods

Two hundred and twenty six bread wheat varieties representative of wheat breeding in Italy from the beginning of XX century up to the present, were selected from the germplasm bank of CREA-SCV. The complete list of the accessions tested, with their pedigree, year of release/registration and collection code is presented in Suppl. material 1. The lines were hand planted in single rows 1.0 m long and 0.40 m apart in S. Angelo Lodigiano (SAL) and Lodi (LO), Italy, during the 2011-2012 and 2012-13 growing seasons. The cultural practices consisted in nitrogen application at tillering (40 kg/ha as ammonium nitrate) and manual control of the weeds. The plants were harvested by hand. The accessions were characterized using 16 morpho-agronomic and qualitative descriptors (Tables 1, 2), including some suggested by the International Board for Plant Genetic Resources (IBPGR 1985). Protein content (PC) (N × 5.7, dry weight, AACC 39-10), and hardness (Ha) (AACC 39-70) (AACC International 2000) were determined on wholemeal by a NIR System Model 6500 (FOSS NIRSystems, Laurel, MD). The SDS sedimentation volume (SSV) was measured on wholemeal according to Preston et al. 1982. Specific SDS Sedimentation Volume (sSSV) was calculated as the ratio between SSV and PC. The morphological descriptors were collected on five plants for each line. The qualitative descriptors were measured from two technical replicates. Statistical analyses were carried out using PAST (Hammer et al. 2001)

## Data resources

The data obtained by the morpho-physiological and qualitative analyses are reported in Suppl. material 2.

## Results

**Figure 1.**
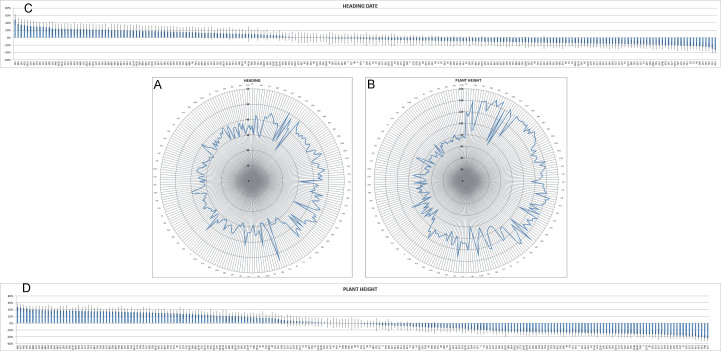
Radar chart of the average heading date (A) and plant height (B) of the lines under study across two locations (SAL and LO) and two growing seasons (2011-12 and 2012-13). The data behind the graphs are reported in Suppl. material 2; only one every four lines is labelled in the graphs. The lines are sorted by year of release. Fold change variation of heading date (C) and plant height (D) is expressed as ratio between the average value of the single line and that of the population. Standard errors (SE) are represented by bars.

**Figure 2.**
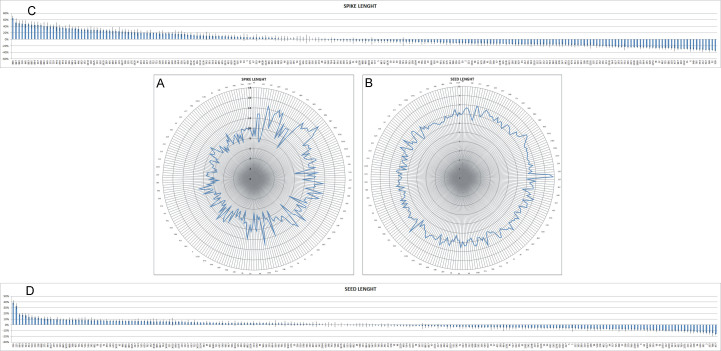
Radar chart of the average spike lenght (A) and seed lenght (B) of the lines under study across two locations (SAL and LO) and two growing seasons (2011-12 and 2012-13). The data behind the graphs are reported in Suppl. material 2; only one every four lines is labelled in the graphs. The lines are sorted by year of release. Fold change variation of spike lenght (C) and seed lenght (D) is expressed as ratio between the average value of the single line and that of the population. Standard errors (SE) are represented by bars.

**Figure 3.**
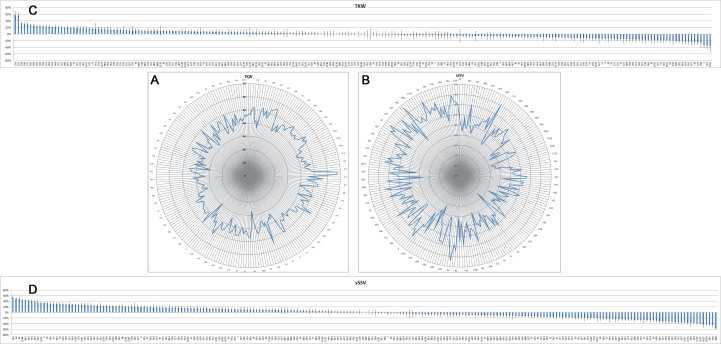
Radar chart of the average thousand kernel weight (A) and specific sedimentation volume (B) of the lines under study across two locations (SAL and LO) and two growing seasons (2011-12 and 2012-13). The data behind the graphs are reported in Suppl. material 2; only one every four lines is labelled in the graphs. The lines are sorted by year of release. Fold change variation of thousand kernel weight (C) and specific sedimentation volume (D) is expressed as ratio between the average value of the single line and that of the population. Standard errors (SE) are represented by bars.

Mean, minimum and maximum values and standard deviations for nine continuously variable descriptors are reported in Table 1, while their frequency distributions are shown in Figs 1, 2, 3. The variability of the descriptors showing discontinuous variation is presented in Table 2. The majority of the lines (62.0%) had erect growth habit of young plants (Table 2); the lines with prostrate growth habit were some landraces and old cultivars, with the exception of Isengrain (released in 1997) and Altamira (2009). Heading date was very variable, with a difference of 30 days between the earliest and the latest accessions and a fold change (FC) variation, with respect to the population average, spanning from +49% to -33% (Table 1 and Fig. 1C). A trend towards earliness was observed going from the oldest to the most recent accessions (Fig. 1A) even if both the latest (Balilla, 55 days) and the earliest (Morru Canu, 25 days) date back to the beginning of the 20^th^ century. Plant height showed a very high variation, spanning from 58 to 153.5 cm, with an average of 103.8 cm and FC going from +47% to -44% (Table 1 and Fig. 1D). A strong decline of plant height was observed over time, with the modern cultivars being much shorter than the older ones (Fig. 1B).

As far as the spike is concerned, the most common shape was oblong, present in 63.8% of the lines, followed by fusiform (26.5%), while only 9.6% of the lines were clavate (Table 2). The length of the spike gradually decreased over time, even if a great variability is present, especially during the first half of the 20th century (Fig. 2 A, C): as a consequence, the extreme values were observed in ancient lines, the landrace Rieti (16 cm) and the cv Impeto, released in 1939 (6.5 cm). As regards spike density, 70.3% of the lines were lax, 18.5% very lax, 10.4% intermediate; very few lines (0.8%) were dense (Table 2), with a tendency towards density increase going from landraces to the new varieties (data not shown). The average number of spikelets per spike was 19, ranging from 15 to 23.8 (Table 1). The lines without awns and with awns longer than 40 mm were the most represented (41.6% and 48.2%, respectively), while both awnletted lines (with 10-40 mm long awns) and lines with very long awns (awns longer than spike) were much less frequent (Table 2). The majority of the lines had white glumes (74.5%) and red seeds (83.4%) (Table 2). The number of seeds per spike averaged 49.2, varying from 30.5 to 66.9 (Table 1). Seed length was only moderately variable, from 6.0 to 9.9 mm (FC from +38% to -16%), and a trend towars a slight length reduction was observed over time (Table 1, Fig. 2 B, D). 1000-kernel weight, on the contrary, was extremely variable, from 27.1 to 67.5 g (FC from +57% to -37%), and a slight reduction was observed going from the ancient lines to the more recent ones (Table 1, Fig. 3 A, C). On average, the seed had a length of 7.1 mm and a weight of 43 mg (Table 1). Soft and medium hardness of the kernel were almost equally represented, while only 8.9% of the lines had hard kernel texture (Table 2). The protein content (PC) varied between 11.3% and 17.4%, with an average of 14.1% and the SDS sedimentation volume (SSV), an indicator of gluten quality, from 17.5 to 61.7 mL (39.9 mL on average) (Table 1). When factoring the protein content into quality assessment by considering the specific sedimentation volume (sSSV: SSV/PC), the FC variation ranged from +51% to -60% and the average value of the parameter was 2.9 (Fig. 3 D and Table 1). A clear trend towards increasing quality was observed from the ancient lines to the modern ones (Fig. 3 B).

The correlations among some analysed variables are reported in Table 3. Most of the correlations were highly significant (P<0.01) due to the large number of observations, hence only values above 0.50 will be discussed here. HD was positively correlated with SL (r= 0.69), PH (r= 0.68), and PC (r= 0.51). PH was positively correlated to SL (r= 0.73), PC (r= 0.67), SeL (r= 0.59) and TKW (r= 0.56), and negatively correlated to Se/S and sSSV (r=- 0.51 for both). SL was positively correlated to PC (r= 0.52), while Se/S was negatively correlated to SeL (r=- 0.56) and TKW (r=- 0.52). Positive correlations were also found between SeL and TKW (r= 0.75) and SSV and sSSV (r= 0.90).

## Discussion

A broad variation for several morpho-physiological and qualitative traits was observed in the bread wheat collection under study, allowing to follow the plant ideotype changing across the breeding progress in Italy during the 20th century.

A strong reduction of plant height was achieved as early as 1914, with the first cultivars released by Nazareno Strampelli. Plant size reduction was mainly achieved by using the short-straw bread wheat Japanese variety Akakomugi, adopted by Nazareno Strampelli in his very first crosses. Strampelli obtained a free sample of seeds in 1911 from the Ingegnoli company in Milan and soon realised it had no agronomic value (Salvi et al. 2012). Nevertheless, the variety possessed two unique traits: it was short-strawed and extremely early maturing. Only *a posteriori* it was demonstrated that Akakomugi is the donor of the reduced height gene *Rht8* and the daylight-insensitive gene *Ppd-D1*, closely linked on chromosome 2D (Borojevic and Borojevic 2005). Due to this linkage, the new varieties simultaneously achieved also earliness. It is interesting to stress that breeding for short straw acquired foremost importance only when the agronomic practices changed, and in particular after the second world war, when mineral fertilizers became largely available. Therefore the genotypes released between 1905 and 1938 were shorter than the landraces and their selections, but only from the 1950s onwards a drastic decrease of plant height can be observed. During the last decade, a slight increase of plant height was registered, mainly due to the adoption of French genotypes which are, on average, taller and slightly later in ripening than their Italian counterparts. Reports showing the reduction of wheat plant height during the 20^th^ century have been published from virtually all countries worldwide, from the United States (Donmez et al. 2001) to the UK (Austin et al. 1980), Pakistan (Parveen and Khalil 2011), Cina (Zhou et al. 2007), Scandinavia (Diederichsen et al. 2013), France (Brancourt-Hulmel et al. 2003), Spain (Sanchez-Garcia et al. 2012), and also Italy (Canevara et al. 1994, De Vita et al. 2007, Guarda et al. 2004). This shift provided a big yield improvement by changing the harvest index, i.e. the proportion of plant biomass in the harvested grain, and introducing short stiff stems that protect against lodging (Langridge 2014).

The spike, over time, became shorter and denser, with more and smaller seeds. All over continental Europe, in the late 19^th^ and early 20th century, the density of the spike was improved by crossing local winter wheat landraces with square head wheats from England (Diederichsen et al. 2013). As suggested by these authors, perhaps in traditional agriculture the loose spikes were an advantage during the final maturing stages, because the air could better move through the bundled plants left in the field to dry. The more recent use of combine harvesters allows the ripening of the spikes on free standing plants, with good air circulation, so loose spikes are not required in modern cultivars.

In our collection, the prostrate growth habit of young plants was far less common than the erect growth habit, and was spotted only in the landrace Solina and in two selections from the landrace Inallettabile. The same situation is reported by Diederichsen et al. 2013), who found just four landraces showing the prostrate habit, along with a larger proportion of plant with intermediate growth habit in landraces than in cultivars.

The majority of the genotypes had red seeds. White wheat usually shows a higher tendency than red wheat to pre-harvest sprouting (Gale 1989): as a consequence, rainy weather during ripening induces the initiation of *in situ* germination, with a concomitant release of amylase enzymes, leading to a loss of test weight and diminished processing quality. The scarcity of white genotypes is therefore probably due to the selection applied by the breeders who discarded sprouted lines, unconsciously eliminating light-coloured genotypes.

As far as the “bread making” quality is concerned, a reduction of the protein content (one of its key factors) was observed going from the landraces to the more recent cultivars. Nevertheless, the quality progressively increased over time, as shown by the increase of the SDS sedimentation volume and of the specific SDS sedimentation volume. Protein content alone, in fact, does not necessarily lead to good bread making quality. In fact, this trait is strongly affected by the allelic composition of the endosperm storage proteins (gliadins and glutenins), which largely inﬂuence strength and elasticity of the dough. In particular, by the middle of the 80s, the results of extensive genetic and biochemical studies clearly demonstrated that differences in the number and type of HMW glutenin subunits strongly affect the breadmaking properties through effects on the amount and size distribution of glutenin polymers. More recently, the contribution of LMW glutenin subunits both *per se* and for their interactions with HMW through additive and epistatic effects became also evident (a thorough dissertation on gluten proteins and their role in breadmaking is present in Shewry et al. 2003). The huge amount of genetical and biochemical studies on wheat storage proteins, gliadins and glutenins, which started in the 80s, was the base for these achievements, leading the breeders to the selection of alleles associated with better technological quality.

Along with quality improvement, a sharp increase in kernel hardness can be observed among the cultivars released since 1970, which show values typical of medium and hard classes. Nevertheless, such trend cannot be ascribed to a precise breeders’ intent, but is a by-product of the selection for high breadmaking quality. In fact wheats with higher kernel texture, although requiring more energy and time for milling, produce flours with optimal technological characteristics for the production of bread and leavened baked products (Carson and Edwards 2009).

The significant positive correlation of heading date with plant height clearly derived from the close linkage between *Rht8* and *Ppd-D1* gene above discussed. On the other end, most of the significant correlations are probably linked to the germplasm composition: the oldest varieties are taller than the most recent ones, and have longer but lax spikes which bear less seeds per spike. As a consequence, the photosynthates accumulation leads to bigger, heavier seeds. Additionally, the oldest varieties have higher protein content but show lower bread making quality, in term of sedimentation volume: hence the significant negative correlation with the specific sedimentation volume.

## Supplementary Material

Supplementary material 1 List of the materials, pedigree, year of release or registration and collection codes Data type: pedegree
File: oo_49791.xls P. Laino, M. Limonta, D. Gerna, P. Vaccino

Supplementary material 2 Morpho-physiological and qualitative data obtained from the lines under study across two locations (SAL and LO) and two growing seasons (2011-12 and 2012-13). Data type: Morpho-physiological and qualitative
File: oo_38659.xls P. Laino, M. Limonta, D. Gerna, P. Vaccino

## Figures and Tables

**Table 1.** Mean, minimum and maximum values and standard deviations for nine continuously variable descriptors measured on the bread wheat accessions under study. The reported data are the average values from two locations (SAL and LO) and two growing seasons (2011-12 and 2012-13).

**Descriptor**	**Code**	**Mean**	**Minimum**	**Maximum**	**Standard deviation**
Heading date (days from 1st April)	HD	37.0	24.8	55.0	5.99
Plant height excluding awns (cm)	PH	103.8	58.0	153.5	27.29
Spike length (cm)	SL	10.0	6.5	16.0	2.08
Spikelets/spike (n°)	Sp/S	19.0	15.0	23.8	1.46
Seeds/spike (n°)	Se/S	49.2	30.5	66.9	7.59
Seed length (mm)	SeL	7.1	6.0	9.9	0.52
1000-kernel weight (g)	TKW	43.0	27.1	67.5	5.89
Protein content (% dm)	PC	14.1	11.3	17.4	1.30
SDS sedimentation volume (mL)	SSV	39.9	17.5	61.7	7.85
Specific SDS sedimentation volume	sSSV	2.9	1.2	4.3	0.60

**Table 2.** Frequency distribution (%) for the discontinuously variable descriptors measured on the bread wheat accessions under study. The reported data are the average values from two locations (SAL and LO) and two growing seasons (2011-12 and 2012-13).

**Descriptor**	**Code**	**Class**	**1**	**2**	**3**	**4**
Growth habit, juvenile	GH	1. Erect 2. Semi-erect 3. Prostrate	62.0	31.0	6.9	
Spike shape	SS	1. Fusiform 2. Clavate 3. Oblong	26.5	9.6	63.8	
Spike density*	SD	1. Very lax (<16) 2. Lax (16-25) 3. Intermediate (26-30) 4. Dense (31-40)	18.5	70.3	10.4	0.8
Awnedness	AW	1. None or <10 mm long awns 2. 10-40 mm long awns 3. Awns longer than 40 mm 4. Awns longer than spike	41.6	4.2	48.2	5.9
Glume colour	GC	1. Red to brown 2. White/amber	25.5	74.5		
Seed colour	SeC	1. Red to brown 2. White/amber	83.4	16.6		
Kernel hardness	Ha	1. Soft 2. Medium 3. Hard	50.4	40.7	8.9	
*n° spikelets per 10 cm length of spike						

**Table 3.** Correlations among traits measured on the bread wheat accessions under study.

	**HD**	**PH**	**SL**	**Sp/S**	**Se/S**	**SeL**	**TKW**	**PC**	**SSV**
**PH**	0.68**								
**SL**	0.69**	0.73**							
**Sp/S**	0.33**	0.11	0.40**						
**Se/S**	-0.30**	-0.51**	-0.26**	0.40**					
**SeL**	0.37**	0.59**	0.50**	-0.12	-0.56**				
**TKW**	0.28**	0.56**	0.44**	-0.07	-0.52**	0.75**			
**PC**	0.51**	0.67**	0.52**	-0.02	-0.46**	0.43**	0.29**		
**SSV**	-0.11	-0.24**	0.03	0.06	-0.05	-0.10	-0.07	0.07	
**sSSV**	-0.31**	-0.51**	-0.19**	0.07	0.15*	-0.27**	-0.19**	-0.37**	0.90**
Level of statistical significance: * P<0.05; ** P<0.01									
